# Correction: Genetic and metabolomic architecture of variation in diet restriction-mediated lifespan extension in *Drosophila*

**DOI:** 10.1371/journal.pgen.1010199

**Published:** 2022-04-27

**Authors:** Kelly Jin, Kenneth A. Wilson, Jennifer N. Beck, Christopher S. Nelson, George W. Brownridge, Benjamin R. Harrison, Danijel Djukovic, Daniel Raftery, Rachel B. Brem, Shiqing Yu, Mathias Drton, Ali Shojaie, Pankaj Kapahi, Daniel Promislow

The authors discovered a coding error in one of the scripts used to generate the R objects for downstream analysis of the dataset used in this article. The error applied a batch correction to the AL (*ad libitum*) portion of the metabolomics dataset, but mistakenly, not the DR (diet-restriction) portion. The error affects the dataset that was normalized “across diets” (the tab labeled “across_diet” in S1 Dataset). The “within_diet” metabolomics data, as well as the lifespan data, are unaffected by this coding error. The affected figures and Supporting information files are detailed below. The authors apologize for the errors. The rest of the figures and primary messages of the paper remain unchanged.

There are errors in Panels A and B of S2 Fig. In Panel A of S2 Fig, the separation of samples by diet in PC (principal component) space should be distinct with respect to both PC1 and PC2, rather than just PC1. In Panel B of S2 Fig, the group of metabolites that change with diet and their corresponding P-values is incorrect. Please view the correct S2 Fig below.

In the Diet specific changes in the metabolome subsection of the Results, there are errors in the second sentence of the third paragraph. The correct sentence is: The first principal component (PC) explained 25.8% and PC2 explained 12.5% of the variation across the entire metabolome, and cleanly separated samples by diet, revealing that DR has a strong effect on the fly metabolome.

In Panel C of Fig 2, the authors present 4 metabolites whose “delta” values are correlated with lifespan response. Upon reanalyzing, the p-values for these results are no longer significant. Please see the correct Fig 2 here.

**Fig 2:**
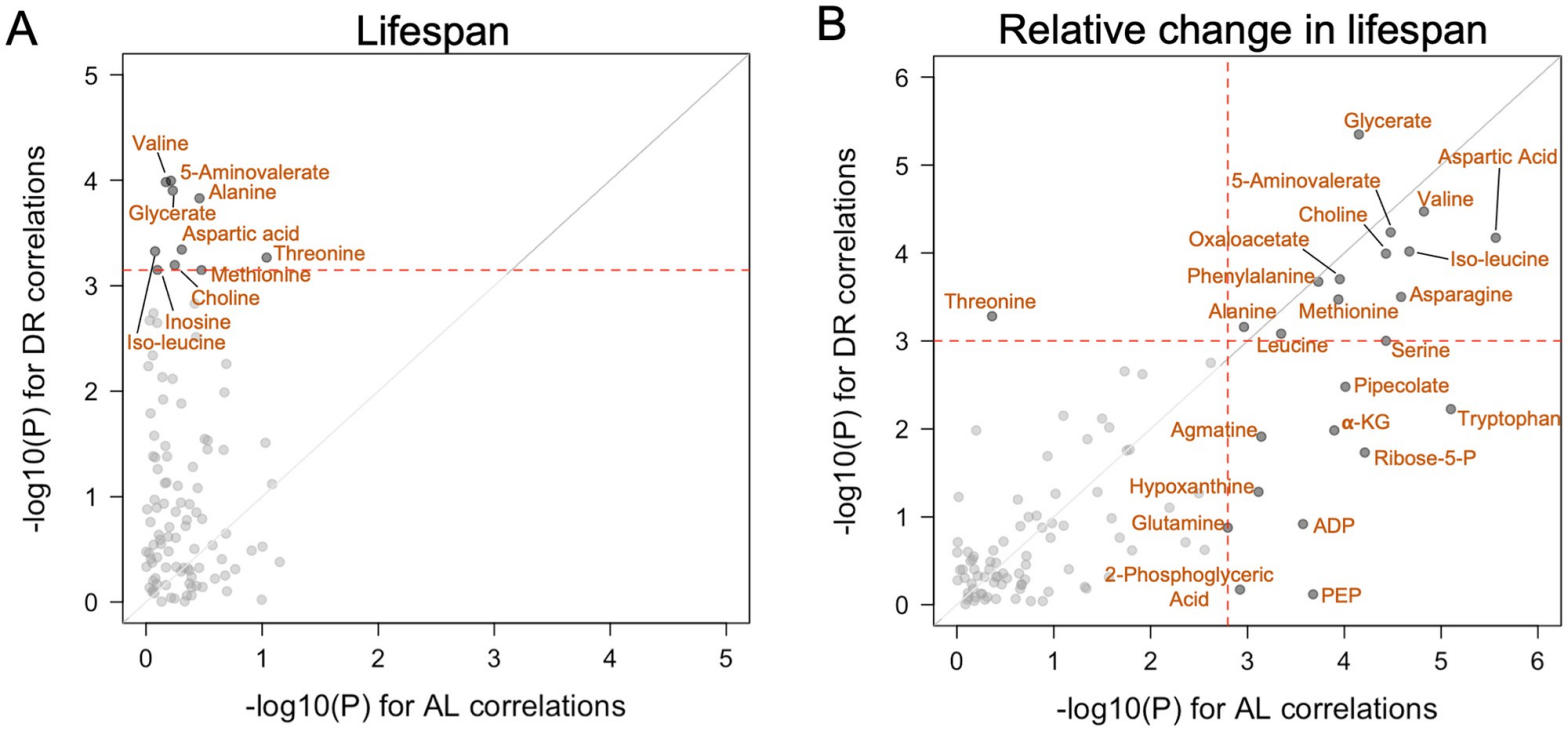
Metabolites significantly correlated with lifespan response. (A,B) Results of univariate analysis modeling lifespan phenotypes as functions of individual metabolites measured under either AL or DR were performed and -log_10_(p-values) were plotted. Each point represents a single metabolite and the significance of its association with (A) mean lifespan and (B) rLS. Red dotted lines represent FDR cutoff at α = 0.01.

In the Metabolites are significantly correlated with lifespan response subsection of the Results, the authors retract the third sentence of the last paragraph.

In the Discussion, there is an error in the eighth sentence of the third paragraph. The correct sentence is: Our study, which is the first that we know of to use metabolome profiling to investigate lifespan response to DR across genotypes, identified 24 metabolites that were correlated with lifespan response either through their baseline abundance or change in abundance across the two diets (Fig 2B and 2C).

There are errors in Tables A and D in S1 Table. Please view the correct S1 Table below.

There are errors in the across_diet section of S1 Dataset. Please view the correct S1 Dataset below.

There are errors in the Data_for_S2 section of S2 Dataset. Please view the correct S2 Dataset below.

## Supporting information

S2 Fig Diet restriction dramatically remodels the metabolome. (A) PCA of all samples using metabolite profiles colored by diet. (B) Volcano plot of significance of the difference between metabolite abundance with DR reveals that almost all metabolites are highly significantly changed with DR. Each point on the plot represents a single metabolite result from a pairwise Student’s t-test.
(PDF)

S1 Table All supplemental tables. (XLSX)

S1 Dataset Normalized metabolome data (within-diet and across-diet normalized data both included). (CSV)

S2 Dataset Numerical data and summary statistics used to make main and supplementary figures. (XLSX)
