# Supplementary material for: Genetic and metabolomic architecture of variation in diet restriction-mediated lifespan extension in Drosophila
Source: PLoS Genet. 2020 Jul 9;16(7):e1008835. doi: 10.1371/journal.pgen.1008835 (PMC7347105; doi:10.1371/journal.pgen.1008835)
Supplement: S1 Fig — (A) Mean lifespan of 161 DGRP lines on AL (5% yeast extract) or DR (0.5% yeast extract) diets. Least squares linear regression of DR lifespan (B) and change in lifespan (DR–AL; C) and rLS (D) as a function of AL lifespan. (PDF) [file pgen.1008835.s001.pdf]

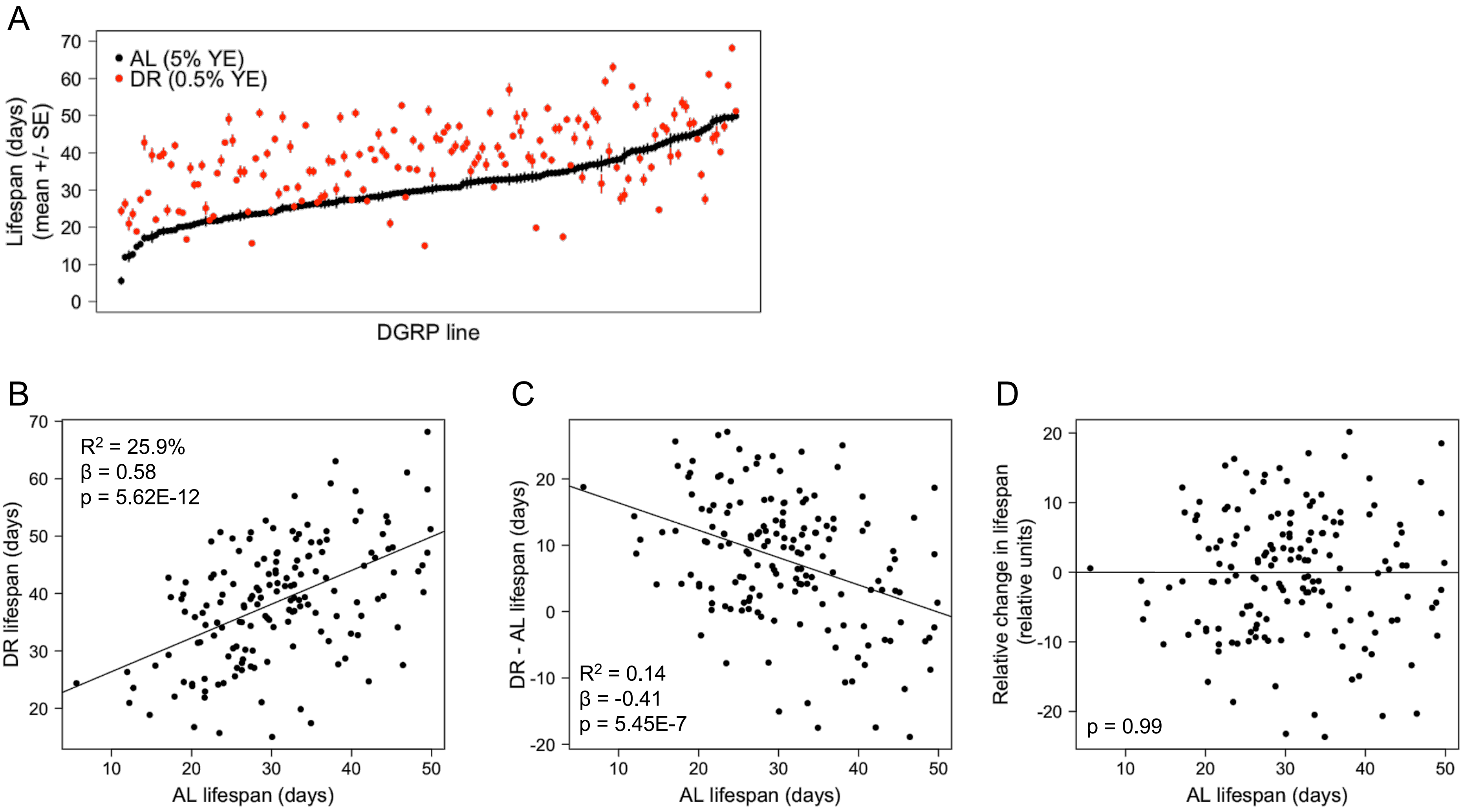

S1 Fig. DGRP lifespan on AL and DR replotted from Wilson *et al*, 2020. (A) Mean lifespan of 161 DGRP lines on AL (5% yeast extract) or DR (0.5% yeast extract) diets. Least squares linear regression of DR lifespan (B) and change in lifespan (DR – AL; C) and rLS (D) as a function of AL lifespan.
