# Supplementary figures and images for: Genetic and metabolomic architecture of variation in diet restriction-mediated lifespan extension in Drosophila
Source: PLoS Genet. 2020 Jul 9;16(7):e1008835. doi: 10.1371/journal.pgen.1008835 (PMC7347105; doi:10.1371/journal.pgen.1008835)

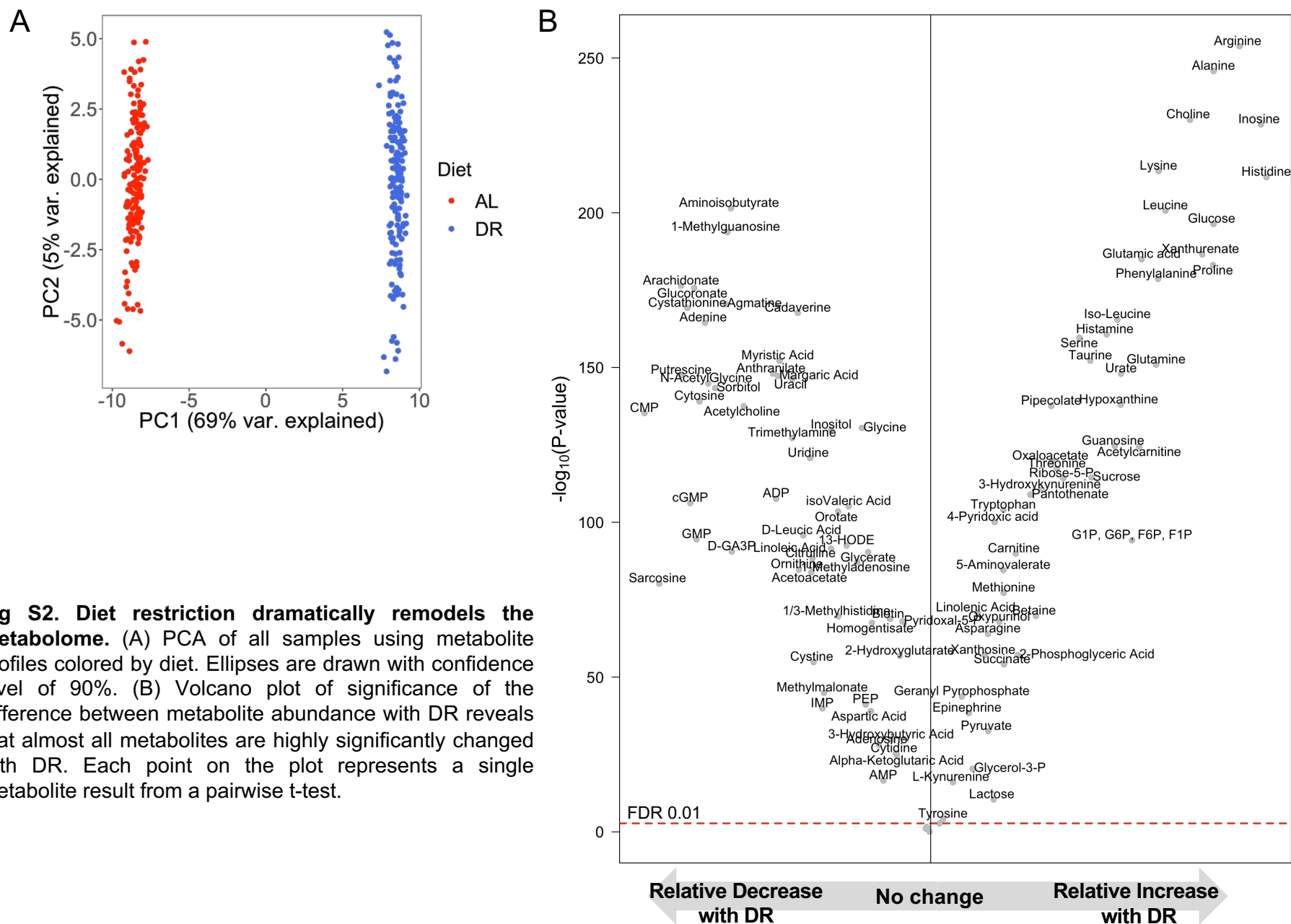

Supplement: S2 Fig — (A) PCA of all samples using metabolite profiles colored by diet. Ellipses are drawn with confidence level of 90%. (B) Volcano plot of significance of the difference between metabolite abundance with DR reveals that almost all metabolites are highly significantly changed with DR. Each point on the plot represents a single metabolite result from a pairwise Student’s t-test. (PDF) [file pgen.1008835.s002.pdf]
