# Supplementary material for: Genetic and metabolomic architecture of variation in diet restriction-mediated lifespan extension in Drosophila
Source: PLoS Genet. 2020 Jul 9;16(7):e1008835. doi: 10.1371/journal.pgen.1008835 (PMC7347105; doi:10.1371/journal.pgen.1008835)
Supplement: S3 Fig — (A) The α-KG/glutamine pathway was manipulated by inhibiting transcript levels of gs1/2 and gdh. (B-G) Survival of RNAi (+RU486) versus control (-RU486) flies of GS2, GS1, and GDH RNAi in whole-body (act5c-gal4-gs driver; B-D), GS2 RNAi in brain (elaV-gal4-gs driver; E), GS2 RNAi in fat body (S106-gal4-gs; F), and GS2 RNAi in gut (5966-gal4-gs; G) on AL and DR. Vertical lines indicate mean survival. Statistical model is a Cox Proportional Hazards model fitting survival as a function of diet, RNAi, and the interaction between diet and RNAi. Hazard ratios (HR) and P values are specific to the interaction term. (PDF) [file pgen.1008835.s003.pdf]

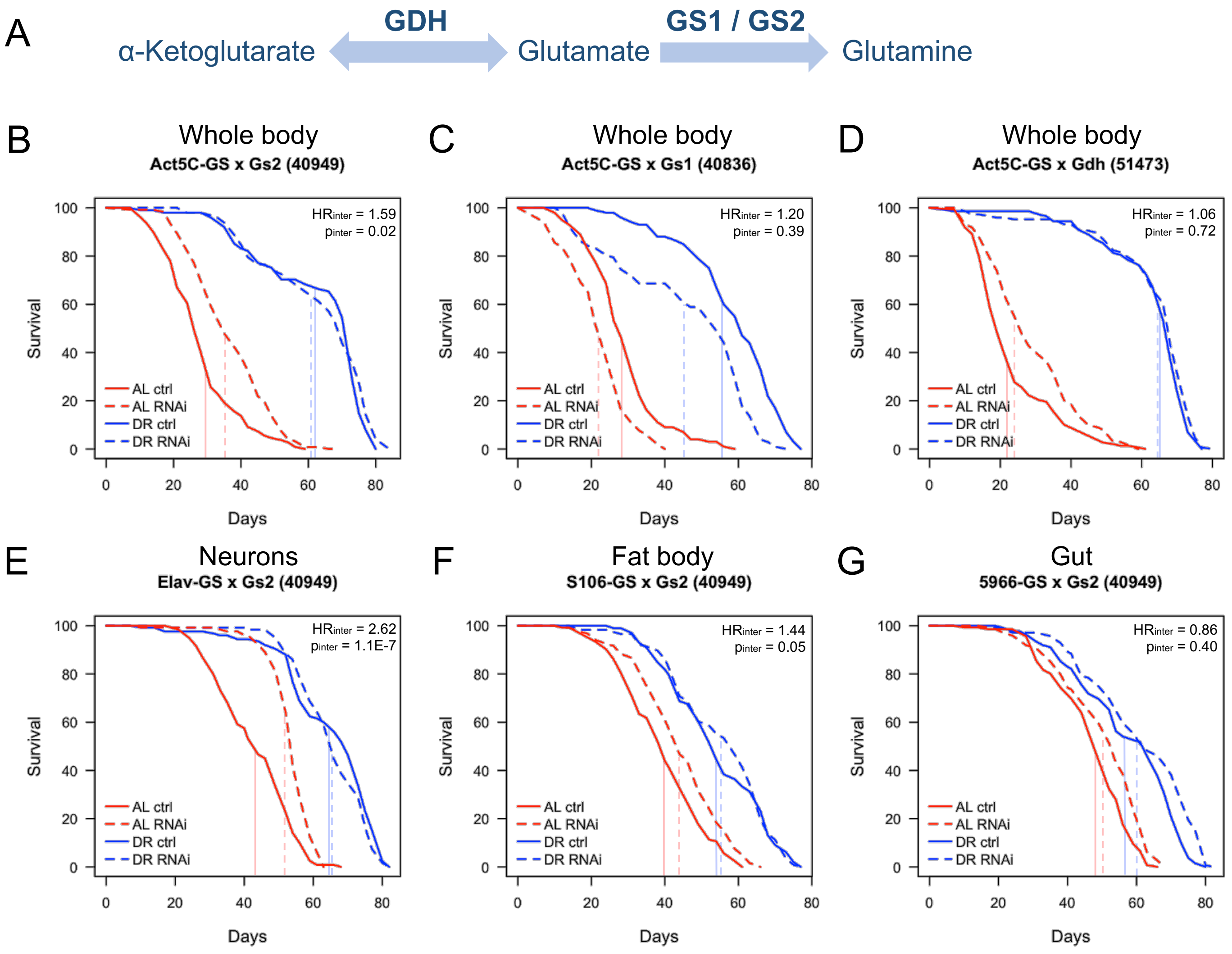

S3 Fig. Diet-dependent survival of GDH, GS1, and GS2 RNAi flies. (A) The α-KG/glutamine pathway was manipulated by inhibiting transcript levels of *gs1/2* and *gdh*. (B-G) Survival of RNAi (+RU486) versus control (-RU486) flies of GS2, GS1, and GDH RNAi in whole-body (act5c-gal4-gs driver; B-D), GS2 RNAi in brain (elaV-gal4-gs driver; E), GS2 RNAi in fat body (S106-gal4-gs; F), and GS2 RNAi in gut (5966-gal4-gs; G) on AL and DR. Vertical lines indicate mean survival. Statistical model is a Cox Proportional Hazards model fitting survival as a function of diet, RNAi, and the interaction between diet and RNAi. Hazard ratios (HR) and p-values are specific to the interaction term.
