# Supplementary material for: Genetic and metabolomic architecture of variation in diet restriction-mediated lifespan extension in Drosophila
Source: PLoS Genet. 2020 Jul 9;16(7):e1008835. doi: 10.1371/journal.pgen.1008835 (PMC7347105; doi:10.1371/journal.pgen.1008835)
Supplement: S4 Fig — Gene-metabolite-phenotype network was constructed from linear modeling and GWAS results from DR metabolites that were correlated with lifespan response as measured by rLS. Gene nodes are colored in teal, metabolite nodes are colored in yellow. Gene node size is directly proportional to node degree, while metabolite node size is held constant. An edge exists between a metabolite and lifespan response if the metabolite was significantly correlated with rLS at a 1% FDR level of significance. Grey labels are metabolites correlated with rLS with no significant gene associations. An edge exists between a gene and metabolite and/or lifespan response if it the gene had a score of ≤1E-4.5. (PDF) [file pgen.1008835.s004.pdf]

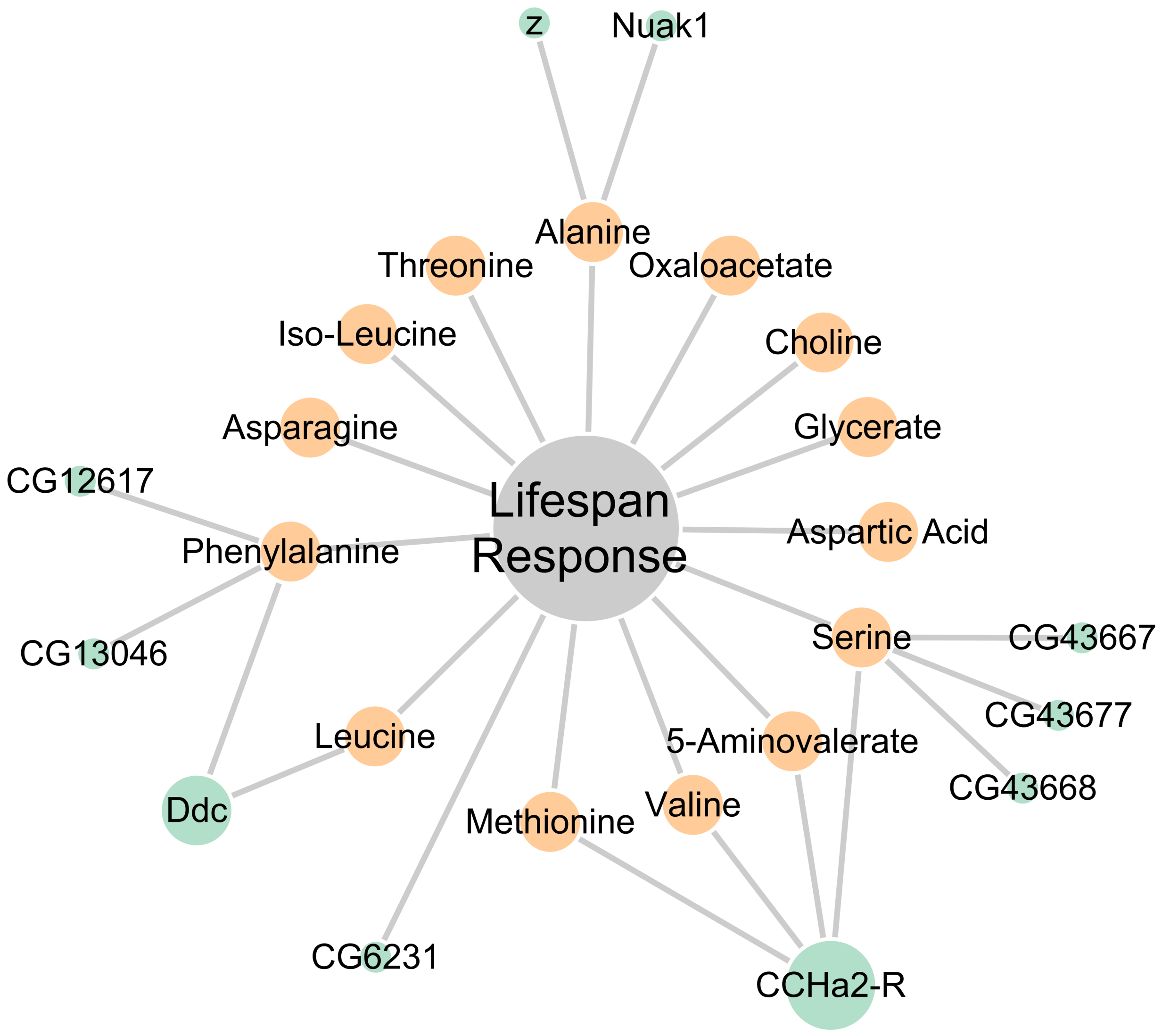

Fig S4. Multi-omic network for lifespan response for DR metabolites. Gene-metabolite-phenotype network was constructed from linear modeling and GWAS results from DR metabolites that were correlated with lifespan response as measured by rLS. Gene nodes are colored in teal, metabolite nodes are colored in yellow. Gene node size is directly proportional to node degree, while metabolite node size is held constant. An edge exists between a metabolite and lifespan response if the metabolite was significantly correlated with rLS at a 1% FDR level of significance. Grey labels are metabolites correlated with rLS with no significant gene associations. An edge exists between a gene and metabolite and/or lifespan response if it the gene had a score of ≤1E-4.5.
