# Supplementary material for: Genetic and metabolomic architecture of variation in diet restriction-mediated lifespan extension in Drosophila
Source: PLoS Genet. 2020 Jul 9;16(7):e1008835. doi: 10.1371/journal.pgen.1008835 (PMC7347105; doi:10.1371/journal.pgen.1008835)
Supplement: S5 Fig — Survival of inducible RNAi (+RU486) versus control (-RU486) flies of selT RNAi. Vertical lines indicate mean survival. Statistical model is a Cox Proportional Hazards model fitting survival as a function of diet, RNAi, and the interaction between diet and RNAi. Hazard ratios (HR) and P values are specific to the interaction term. (PDF) [file pgen.1008835.s005.pdf]

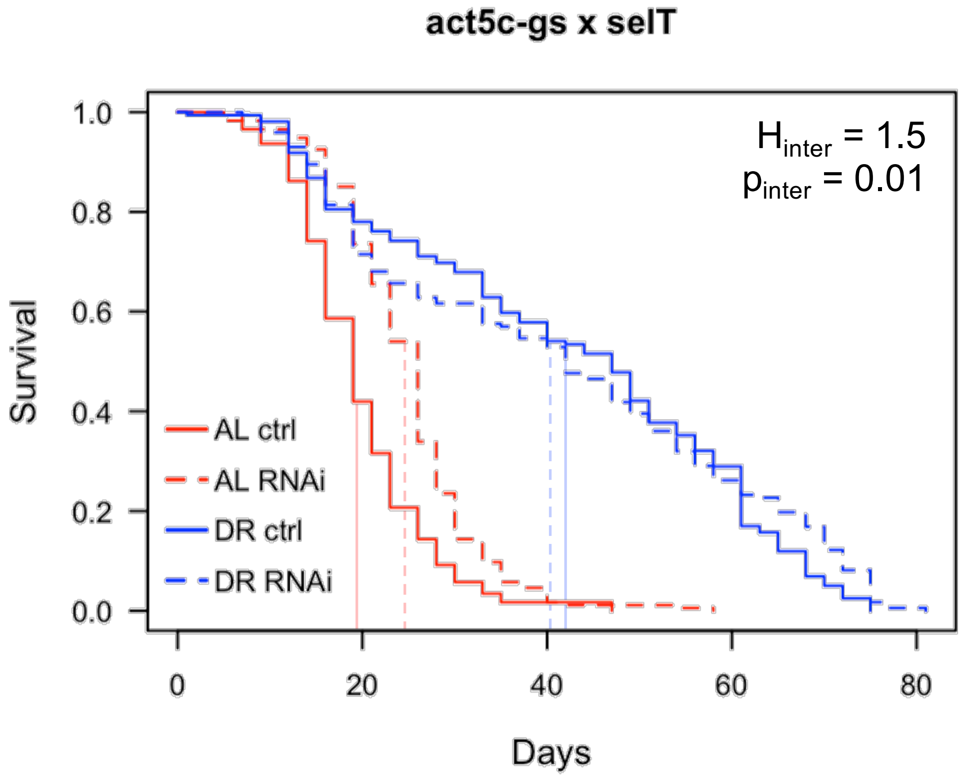

S5 Fig. Diet-dependent survival of RNAi fly strains. Survival of inducible RNAi (+RU486) versus control (-RU486) flies of *selT* RNAi. Vertical lines indicate mean survival. Statistical model is a Cox Proportional Hazards model fitting survival as a function of diet, RNAi, and the interaction between diet and RNAi. Hazard ratios (HR) and p-values are specific to the interaction term.
