# Supplementary material for: Genetic and metabolomic architecture of variation in diet restriction-mediated lifespan extension in Drosophila
Source: PLoS Genet. 2020 Jul 9;16(7):e1008835. doi: 10.1371/journal.pgen.1008835 (PMC7347105; doi:10.1371/journal.pgen.1008835)
Supplement: S1 Text — (PDF) [file pgen.1008835.s009.pdf]

Supplementary Methods

Metabolomics Sample Preparation

Frozen fly samples were thawed at room temperature for 45 min and subsequently prepared following previously described procedure [1-3]. Samples were homogenized in 200 µL of 10:1 PBS:Water, 800 µL of methanol containing known concentrations of 6C13-glucose and 2C13-glutamate was added, samples were vortexed for 10 seconds and stored at -20 °C for 20 min. Afterwards, samples were sonicated in an ice bath for 10 min, centrifuged at 15,000 rpm for 15 min. at 4 °C, and 600 µL of supernatant was recovered and transferred to a new Eppendorf tube. At the end, samples were dried using the SpeedVac drier for 2h at 30 °C, and dried samples were reconstituted in 10 mM ammonium acetate in 30% H_2O/ 65% acetonitrile/ 5% methanol + 0.3 % acetic acid containing known concentrations of 2C13-Tyrosine and 1C13-Lactate. The reconstituted samples were vortexed and centrifuged again and 300 µL of supernatant was recovered and transferred into LC vial. At this point, samples were ready for LC-MS analysis. Four stable isotope-labeled internal standards (13C-lactate, 13C-tyrosine, 13C-glucose and 13C-glutamic acid were added to each sample in two separate sample prep steps in order to monitor the system performance and sample prep accuracy. Two sets of quality control (QC) samples were used to monitor the assay performance and data reproducibility. Internal QC (QC-I) was a pooled serum samples that was routinely used by The NWMRC to monitor the assay performance and the sample QC (QC-S) was made by pooling randomly chosen 30 fly samples and this QC was used to monitor the data reproducibility.

Liquid Chromatography (LC)

The LC system was composed of two Agilent 1260 binary pumps, an Agilent 1260 auto-sampler and Agilent 1290 column compartment containing a column-switching valve (Agilent Technologies, Santa Clara, CA). The LC modules were controlled by Analyst 1.5 software (AB Sciex, Toronto, ON, Canada). Each sample was injected twice, 15 µL for analysis using negative ionization mode and 5 µL for analysis using positive ionization mode. Both chromatographic separations were performed in HILIC mode on two XBridge BEH Amide columns (150 x 2.1 mm, 2.5 µm particle size, Waters Corporation, Milford, MA, Part No. 186006724). While one column was performing the separation, the other column was getting reconditioned and ready for the next injection. The flow rate was 0.300 mL/min, auto-sampler temperature was kept at 4℃, the column compartment was set at 30℃, and total separation time for both ionization modes was 20 min (total analysis time per sample was 40 min). The mobile phase was composed of Solvents A (10 mM ammonium acetate in 90%H_2O/ 5% acetonitrile/ 5% methanol + 0.3% acetic acid) and B (10 mM ammonium acetate in 90% acetonitrile / 10% water + 0.3% acetic acid). The gradient conditions for both separations were as follows: 0-2 min:10%A; 2-6 min: from 10% A to 55% A; 6-10 min: 55% A; 10-12 min: 55% A to 10% A; 12-20 min: 10% A.

Mass Spectrometry (MS)

After the chromatographic separation, MS ionization and data acquisition were performed using an AB Sciex QTrap 5500 mass spectrometer (AB Sciex, Toronto, ON, Canada) equipped with an electrospray ionization (ESI) source. The instrument was controlled by Analyst 1.5 software. Targeted data acquisition was performed in multiple-reaction-monitoring (MRM) mode. We monitored 122 and 84 MRM transitions in negative and positive mode, respectively (206 MRM transitions total corresponding to 204 metabolites and 4 stable isotope-labeled internal standards). The dwell (scan) time for each MRM transition was 4 msec. The source and collision gas was N_2 (99.999% purity). The ion source conditions in negative mode were: Curtain Gas (CUR) = 30 psi, Collision Gas (CAD) = 8 L/min, Ion Spray Voltage (IS) = - 3.8KV, Temperature (TEM) = 600℃, Ion Source Gas 1 (GS1) = 50 psi and Ion Source Gas 2 (GS2) = 40 psi. The ion source conditions in positive mode were: Curtain Gas (CUR) = 30 psi, Collision Gas (CAD) = 8 L/min, Ion Spray Voltage (IS) = 3.8KV, Temperature (TEM) = 600 °C, Ion Source Gas 1 (GS1) = 50 psi and Ion Source Gas 2 (GS2) = 40 psi.

Data Acquisition Sequence

Samples were randomized prior to the sample preparation. The LC-MS data was acquired in the following order: Internal quality control (QC-I), sample quality control (QC-S), 10 study samples, blank, repeat. Columns were washed after 6 sets of QCs and 60 study samples. 76 QC runs (38 QC-I and 38 QC-S) and 368 sample runs were performed. The QC data were used to determine average and median coefficient of variance (CV) for each measured metabolite.

MS Data Processing

After the LC-MS data acquisition, the raw data were processed using MultiQuant 2.1 software (AB Sciex, Toronto, ON, Canada). Relative metabolite concentration was reported as peak area under the MRM curve (the MS signal was recorded as ion counts per second).

Quality Control (QC) Monitoring

The average and median CVs for QC-S were 9.6% and 9.2 %, respectively. The average and median CVs for QC-I were 10% both. The average and median CVs for the four metabolites that had corresponding stable isotope-labeled internal standards (SILISs) were all under 5% for both QC-S and QC-I. The signal intensities of 4 stable isotope-labeled internal standards were used to determine the data reliability for each individual study sample. The 4 SILISs were added in 2 separate sample prep steps. If the average signal of these SILISs in a particular sample was ± 15% of the average signal across all the study samples, then the data for this particular sample was not reliable and was excluded (this

indicated that there was a sample prep error due to an inaccurate solvent volume dispensing, or that the matrix affect was too strong indicating a questionable sample quality). The data for 12 samples were removed following the above described criteria.

References for Supplementary Methods

1. Sperber H, Mathieu J, Wang Y, Ferreccio A, Hesson J, Xu Z, et al. The metabolome regulates the epigenetic landscape during naive-to-primed human embryonic stem cell transition. Nat Cell Biol. 2015;17(12):1523-35. doi: 10.1038/ncb3264. PubMed PMID: 26571212; PubMed Central PMCID: PMCPMC4662931.
2. Du J, Rountree A, Cleghorn WM, Contreras L, Lindsay KJ, Sadilek M, et al. Phototransduction Influences Metabolic Flux and Nucleotide Metabolism in Mouse Retina. J Biol Chem. 2016;291(9):4698-710. doi: 10.1074/jbc.M115.698985. PubMed PMID: 26677218; PubMed Central PMCID: PMCPMC4813492.
3. Chiao YA, Kolwicz SC, Basisty N, Gagnidze A, Zhang J, Gu H, et al. Rapamycin transiently induces mitochondrial remodeling to reprogram energy metabolism in old hearts. Aging (Albany NY). 2016;8(2):314-27. doi: 10.18632/aging.100881. PubMed PMID: 26872208; PubMed Central PMCID: PMCPMC4789585.

Summary Statistics and Explanation of Numerical Data for Figures

Main Figures

Fig 1: The numerical data and summary statistics used to make this figure are included in S2_Dataset.xlsx.

Fig 2: The numerical data and summary statistics used to make this figure are included in Tables B, C, and D in S2_Table.xlsx. The metabolome data used for these linear models are in S1_Dataset.xlsx (within-diet normalized).

Fig 3: The edgelist for this network is included in S2_Dataset.xlsx. The metabolome data for panels B-D are in S1_Dataset.xlsx (within-diet normalized).

Fig 4: The edgelist and summary statistics used to make this figure are included in Table E in S1_Table.xlsx.

Fig 5: The genetic variant calls used to make panels B and C are available online at http://dgrp2.gnets.ncsu.edu/. The metabolome data are in S1_Dataset.xlsx (within-diet normalized). The relative lifespan values are in S2_Dataset.xlsx. The survival data used to make panels E and F are included in S2_Dataset.xlsx.

Supplementary Figures

S1 Fig: The numerical data used to make this figure are included in S2_Dataset.xlsx

S2 Fig: The numerical data used to make panel A is included in S2_Dataset.xlsx and the data used to make panel B is included in Table A in S1_Table.xlsx

S3 Fig: The survival data used to make this figure are included in S2_Dataset.xlsx

S4 Fig: The edgelist and summary statistics used to make this figure are included in Table E in S1_Table.xlsx.

S5 Fig: The survival data used to make this figure are included in S2_Dataset.xlsx
