# Supplementary material for: Genetic and metabolomic architecture of variation in diet restriction-mediated lifespan extension in Drosophila
Source: PLoS Genet. 2020 Jul 9;16(7):e1008835. doi: 10.1371/journal.pgen.1008835 (PMC7347105; doi:10.1371/journal.pgen.1008835)
Supplement: Attachment — Submitted filename: response_to_reviewers_2020-03-18.docx [file pgen.1008835.s010.docx]

Reviewer #1

In the present study, Jin. et al., aim to understand how the response to life-extending interventions varies between genotypes. Previously, dietary restriction (DR) has been shown to extend lifespan in most model organisms. However, studies in yeast, flies and mice demonstrated extensive within-organism variation between varying genetic backgrounds. The mechanism for this difference remains understudy, a key question if basic biology of aging is to be extended to generate usable human therapeutics. Therefore, the authors applied a systems biology approach to build candidate gene-to-metabolite-to-phenotype pathways that may underly the differential response to DR in different strains of inbred flies. The authors identify specific metabolic hubs that explain DR response variation, as well as gene-metabolite pairs strongly associated with DR lifespan extension. Additionally, the authors validated two of their gene hits using RNAi lifespans.

Overall, this study is very timely and provides insight into the mechanisms underlying natural variation in DR response, which will likely open doors for further mechanistic studies. These data will be of interest to both the aging and systems biology fields, along with more broadly those interested in precision medicine type approaches to gene/environment interactions.

>We appreciate the very encouraging feedback.

Main points:

o In the data set used around 30% of the population DR didn’t extend lifespan, around half of these had their lifespan shorten with DR. In addition to using mean lifespan and relative lifespan change as a measurement to capture the overall effect, might it give more detection power to extend the analysis to bins of subgroups with differential effect directionality (i.e. responders, non-responders, responded negatively) and look for significant differences between those binned groups?)

>We did exactly this for metabolite associations and saw similar hits so we decided to include only the quantitative measurements of lifespan to simplify the Results and Discussion.

o The authors did the metabolomics and gene-level GWAS data collection on flies at Day 5 after diet started. I would recommend including a discussion about the timing of this measurement; when is the onset of metabolic changes associated with DR thought to be?

> Thank you for that comment—we have now done so. In previous studies of DR from our group (Laye *et al.* 2015) and others (Avanesov *et al.* 2014), longitudinal metabolomic profiling was performed on AL and DR flies at ages as young as 10 days. Both studies showed distinctive metabolome profiles in AL versus DR flies as measured via PCA at 10 days, and show a similar if not greater effect in older flies (20 or 40 days in Laye *et al.* 2015) as well.

We chose to sample at 5 days after beginning the diet (the flies were approx. 7-9 days old at this time) because we are interested in capturing early metabolomic indicators of DR response. Other studies from our group have also shown non-metabolomic phenotypic changes in response to diet as early at 2 days (Lang *et al*., 2019). Per the reviewer’s suggestion, we have added some discussion about this motivation and additional details of the experimental setup in the Methods:

Edited text on page 15: “Fourteen days after set-up when the experimental flies were estimated to be 2-3 days old, mated female flies were sorted into approximately 8 vials of either high yeast extract food… or low yeast extract food…

…We chose to sample at 5 days to try and capture early metabolomic indicators of DR response. Published studies from our group and others have shown metabolomic and other phenotypic differences in response to DR as early as 2-10 days after beginning the experimental diet [11, 42, 73].

o The authors identified genes (for example, SNP in CCHaR) and metabolites (for example, alpha-ketoglutarate) strongly associated with lifespan, and validated them using RNAi. One of the strengths of the paper is the identification of gene-metabolite interactions that are associated with a phenotype. However, the addition of a targeted metabolomics measurement in the validation experiment could strengthen the conclusions – ie looking at the impact of DR +/- knock down of CCHaR.

> While we completely agree that additional metabolomic information for the RNAi lines, particularly CCHa2R, would be very interesting and informative, it would unfortunately not be feasible to obtain these data within the 30 day response window allotted to us when factoring in the time required to raise flies, measure metabolites, and perform analysis. We also feel that even without it, the primary message of our multi-omic analysis (that we are able to identify biologically meaningful candidate mechanisms) is still effectively conveyed.

Minor points

o In lifespans (Figures 5, S3, S5), it would be beneficial to include the mean lifespan line, and report the effect measurement. The effect seems to be more of an increase in median lifespan versus maximum lifespan; revise when reporting the results and drawing conclusions.

>Thank you for these suggestions. Mean lifespan lines and hazard ratios have been added to the survival figures. We hesitate to emphasize maximum lifespan values given our sample sizes (100-150 flies per condition). Instead we are interested in the diet by RNAi interaction effect as a measure of the effect of that gene on lifespan response, which is emphasized throughout the text.

o Throughout the graphics, the authors should highlight the metabolites and genes that are discussed, to make it clearer to the reader where the hits come from.

>We assume the reviewer is referring to the network figures (Figs 3, 4, S4) for this comment. We have added asterisks to the hub metabolites in Fig 3 that are discussed the most extensively in the text.

We hesitate to add any emphasis to specific metabolites/genes in Figs 4 and S4 because we do not want to give the casual reader who is browsing through figures the impression that the highlighted metabolites or genes are more relevant to DR than the rest – they just happen to be the ones we experimentally validated.

o In page 13, 5th sentence the connector ‘and’ is missing from the phrase ‘peripheral organs the brain’.

>Fixed, thank you.

Reviewer #2

This is a very strong, highly integrative submission dealing with one of the most central problems in the biology of aging: by what mechanism does dietary restriction extend lifespan. The results are uniquely informative. The synthetic, systems biology approach is ground-breaking. Interested readers will span those from the medical sciences, neurobiology, longevity research, nutritional research, computational biology, genetics and systems biology. I find no essential faults with the design or analysis. I think the conclusions are well supported and within the scope of the data. I especially like the validation work where candidate hubs are tested by RNAi manipulative experiments. This is the way to move from GWAS to inference on mechanism. Naturally, I am intrigued by the various hits and proposed regulatory pathways for control of variation in the dietary restriction response. These leads will stimulate substantial research.

>We appreciate the very encouraging feedback.

I have only a few editorial or aside remarks. The hardest problem will involve the ability to generalize the outcomes given the way diet was manipulated. This issue needs community level work.

Please clarify the data usage and relationship with the Wilson manuscript. I cannot tell if the survival data and sampled flies of this PLoS submission are de novo material unique to the PLoS submission, or if these data and flies were actually one and the same as those presented in Wilson. Please clarify as well, that Wilson was uploaded to BioRxiv. I would not call this “originally published” as stated on page 6. Or, perhaps the Wilson work has actually been peer reviewed and ‘published’ by now. Using the flies and data in two papers is fine, but simply would require clarification. Finally, it seems that the Wilson project describes several candidate variant genes to affect diet dependent longevity, yet the present work finds perhaps only one (CG6231).

>The lifespan data are the same as those presented in Wilson *et al* (included with the original submission). The flies used for metabolomic sampling in our study came from the same cohort of flies as those used in Wilson *et al*. The metabolomic data are being published for the first time in our study. Wilson *et al* is currently in the process of revision and resubmission at another journal. In light of the reviewer’s comments, we have added this to the text in the Results:

Addition to page 5: “The flies used for metabolomics were collected from the same cohort of flies used for lifespan measurements. The lifespan measurements across the DGRP on AL and DR were first presented and discussed in Wilson *et al*., currently available on BioRxiv [10], while the metabolomic data are novel to this study.”

The reviewer is correct in that Wilson *et al* finds multiple gene candidates associated with diet-dependent longevity while we found only one. The two manuscripts measure different metrics derived from the survival data. In the case of Wilson *et al.*, for their GWAS they fit a linear model predicting mean lifespan with an interaction term between genotype and diet. In our paper reviewed here, we sought SNPs associated with ” change-in-lifespan” (the residual value from a fit of DR lifespan versus AL lifespan across DGRP lines), and did not include an interaction term. Given that the two models fit two different response variables to different covariates, it does not surprise us that we obtain different results. In light of the reviewer’s comment, we have included discussion of this in our Discussion:

Addition to page 11: “The lifespan results we present in this study were also analyzed by Wilson *et al* [10]. We note that Wilson *et al.* found multiple candidate variants that affect diet-dependent longevity, while we identify one that met our significance cutoff (Fig 4 and S4 Fig). This is not surprising, given that these two studies fit two different response variables to different covariates. Wilson *et al.* used a linear model predicting mean lifespan with an interaction term between genotype and diet for their GWAS, while we use relative lifespan as the outcome for our GWAS.”

On page 9, the text (first paragraph) mentions “significance scores for each gene-metabolite pair”. Readers are referred to the methods for details. I recommend adding a sentence to define these scores here; this will aide in understanding the paragraph without putting the reader in a bind to figure out details on their own in the M&M.

Addition to page 8 (formerly page 9): “Briefly, gene scores were assigned by taking the minimum p-value from all variants associated with a gene after adjustment via at least 10,000 rounds of permutation testing. A detailed explanation of this calculation is included in the Methods.”

The paper acknowledges ‘Limitations of study’. I like this frankness. An important issue, as noted, will be the method used to make diet based on yeast extract. Given this method, the results and conclusions seem very robust and real. The problem will be that most (all) other labs use processed whole yeast in adult diets, not yeast extract, and the authors know about this controversy. It is a positive move to acknowledge the issue, but this does not fix the problem or establish the generality of their mechanistic conclusions. I am not sure what to recommend here but I can see the paper will always have an asterisk until its results are verified using more conventional diets, perhaps limiting these tests to the candidate gene RNAi studies.

>We agree wholeheartedly. Given the novelty of our methods and results, our hope is certainly that this work inspires follow-on studies in other labs, including those using whole yeast diets.

Page 16. Please elaborate and clarify the logic for how residuals are used make the rLS. I don’t understand how residuals ‘remove potentially confounding effects of extreme AL …” Residuals are calculated for all points, and adjusts each observation relative to its expected value based on linear regression. Why is this approach used instead of, perhaps, the ratio of DR to AL median longevity?

>We realize that the original phrasing of “extreme AL” is misleading – our intention was to control for the effects of AL lifespan in general, not just extreme AL lifespan. This has been changed in the text (page 16).

There are numerous problems with simply looking at (DR – AL) or (DR/AL). In the first case, (DR-AL) and AL are autocorrelated, so that we would not be sure that we were identifying correlates of the DR response, versus underlying AL lifespan. In the second case, where the metric is the ratio of DR to AL, the error variance is skewed by the value of the denominator in a non-linear way – as an example: the difference between 50/10 and 50/11 (difference of ~0.5) is much larger than the difference between 50/100 and 50/101 (difference of ~0.01), when in both cases, the difference in the denominator (in the case of our study, we might use AL lifespan) is only 1 day. Residuals from a linear model of DR vs. AL resolve both of these issues.

By definition, taking the residuals of Y relative to X in a least squares regression removes the effect of X from Y. For our purposes of wanting to remove the effects AL lifespan from the value of DR lifespan, the residuals seemed to be an appropriate solution. Fig S1B is the visualization of the model that was used to calculate the residuals (DR lifespan as a function AL lifespan), and Fig S1D is what the “new Y” (or rLS) looks like having removed the effect of AL.
