# Supplementary material for: Correction: Genetic and metabolomic architecture of variation in diet restriction-mediated lifespan extension in Drosophila
Source: PLoS Genet. 2022 Apr 27;18(4):e1010199. doi: 10.1371/journal.pgen.1010199 (PMC9045613; doi:10.1371/journal.pgen.1010199)
Supplement: S2 Fig — (A) PCA of all samples using metabolite profiles colored by diet. (B) Volcano plot of significance of the difference between metabolite abundance with DR reveals that almost all metabolites are highly significantly changed with DR. Each point on the plot represents a single metabolite result from a pairwise Student’s t-test. (PDF) [file pgen.1010199.s001.pdf]

# Supplemental Figure 2

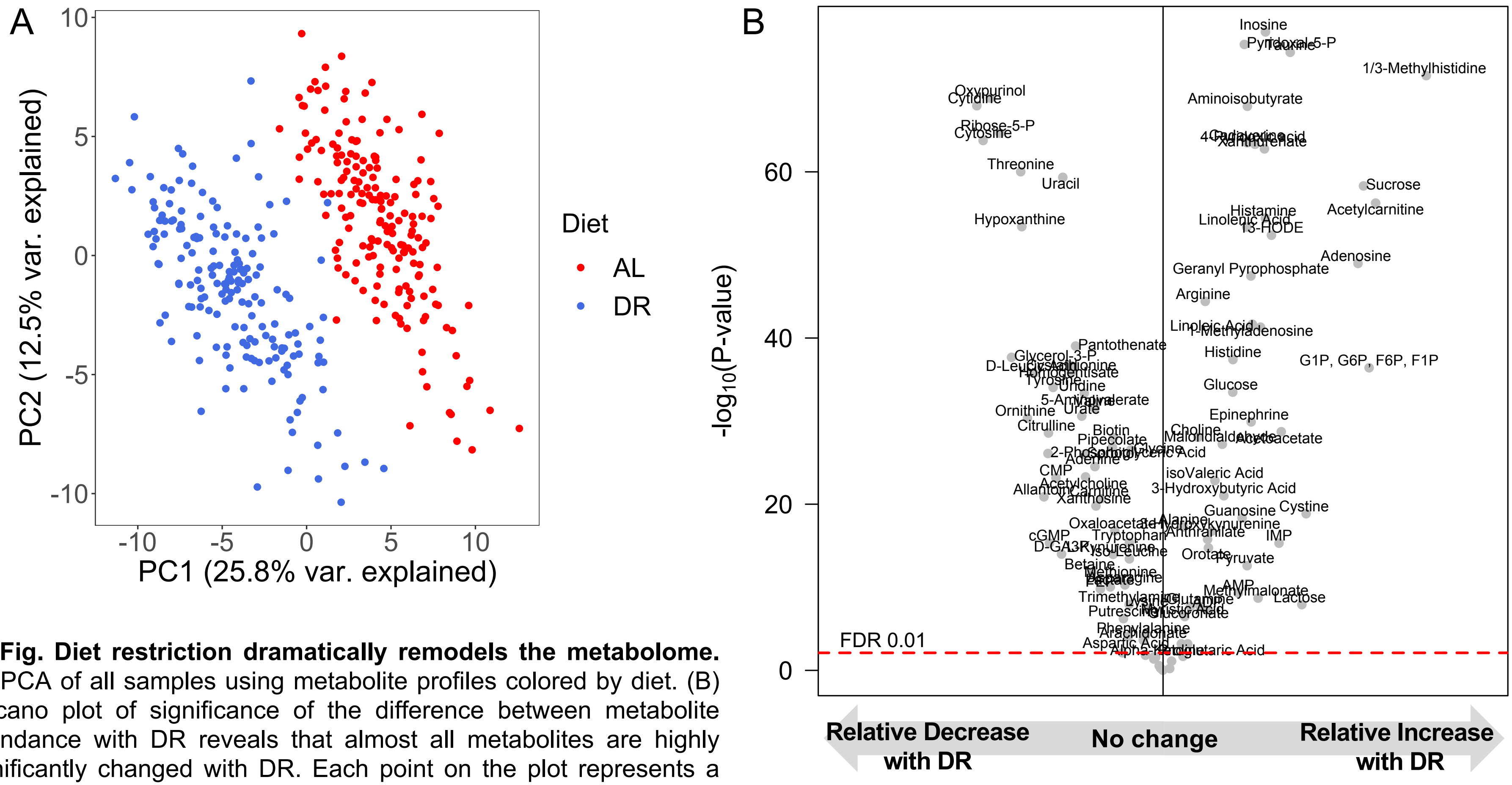


**S2 Fig. Diet restriction dramatically remodels the metabolome.** (A) PCA of all samples using metabolite profiles colored by diet. (B) Volcano plot of significance of the difference between metabolite abundance with DR reveals that almost all metabolites are highly significantly changed with DR. Each point on the plot represents a single metabolite result from a pairwise Student's t-test.
